# Autophagy limits proliferation and glycolytic metabolism in acute myeloid leukemia

**DOI:** 10.1038/cddiscovery.2015.8

**Published:** 2015-08-17

**Authors:** AS Watson, T Riffelmacher, A Stranks, O Williams, J De Boer, K Cain, M MacFarlane, J McGouran, B Kessler, S Khandwala, O Chowdhury, D Puleston, K Phadwal, M Mortensen, D Ferguson, E Soilleux, P Woll, SEW Jacobsen, AK Simon

**Affiliations:** 1 Translational Immunology Lab, BRC- NIHR, John Radcliffe Hospital, Experimental Medicine, Nuffield Department of Medicine, Oxford OX3 9DU, UK; 2 MRC Human Immunology Unit, Weatherall Institute of Molecular Medicine, John Radcliffe Hospital, Oxford OX3 9DS, UK; 3 Molecular Haematology and Cancer Biology Unit, UCL Institute of Child Health, University College London, London WC1N 1EH, UK; 4 MRC Toxicology Unit, Hodgkin Building, University of Leicester, PO Box 138, Lancaster Road, Leicester LE1 9HN, UK; 5 Target Discovery Institute, Nuffield Department of Medicine, University of Oxford, Roosevelt Drive, Oxford OX3 7FZ, UK; 6 Haematopoietic Stem Cell Biology, MRC Molecular Haematology Unit, Weatherall Institute of Molecular Medicine, John Radcliffe Hospital, Oxford OX3 9DS, UK; 7 Nuffield Department of Clinical and Laboratory Sciences, John Radcliffe Hospital, Oxford OX3 9DU, UK

## Abstract

Decreased autophagy contributes to malignancies; however, it is unclear how autophagy has an impact on tumor growth. Acute myeloid leukemia (AML) is an ideal model to address this as (i) patient samples are easily accessible, (ii) the hematopoietic stem and progenitor cells (HSPC) where transformation occurs is well characterized and (iii) loss of the key autophagy gene *Atg7* in HSPCs leads to a lethal pre-leukemic phenotype in mice. Here we demonstrate that loss of *Atg5* results in an identical HSPC phenotype as loss of *Atg7*, confirming a general role for autophagy in HSPC regulation. Compared with more committed/mature hematopoietic cells, healthy human and mouse HSPCs displayed enhanced basal autophagic flux, limiting mitochondrial damage and reactive oxygen species in this long-lived population. Taken together, with our previous findings these data are compatible with autophagy-limiting leukemic transformation. In line with this, autophagy gene losses are found within chromosomal regions that are commonly deleted in human AML. Moreover, human AML blasts showed reduced expression of autophagy genes and displayed decreased autophagic flux with accumulation of unhealthy mitochondria, indicating that deficient autophagy may be beneficial to human AML. Crucially, heterozygous loss of autophagy in an MLL–ENL model of AML led to increased proliferation *in vitro*, a glycolytic shift and more aggressive leukemias *in vivo*. With autophagy gene losses also identified in multiple other malignancies, these findings point to low autophagy, providing a general advantage for tumor growth.

## Introduction

Autophagy is a catabolic delivery pathway for excess or damaged cytoplasmic constituents to the lysosomes where macromolecules are broken down and their components freed for anabolic activities.^1^

Autophagy maintains mitochondrial health and metabolic pathways, being induced following metabolic stress, controlled by mTOR complex 1 (mTORC1). Under favorable conditions, activated mTORC1 signals for cell growth, promoting translation, cell cycle progression and glycolysis^2^ while inhibiting autophagy. To maximize cell mass during proliferation, suppression of self-catabolism may be vital for growth activities. Indeed, induction of autophagy prolongs cell survival at the cost of cell size and growth.^3,4^

Activation of the Akt/mTOR pathway is a common feature of cancers, including leukemias^5^ and is required for proliferation in AML models.^6^ Knockout of autophagy genes in mice is associated with hyperproliferation in some tissues^7,8^ and eventual tumor development.^9^ Our previous studies indicated that mice without the autophagy gene *Atg7* in the hematopoietic system develop pre-leukemic myeloproliferation.^10^ However, it remained unclear how *Atg7* promotes cell proliferation and whether this was an *Atg7*-specific function.^11^ Despite oncogenic effects in these models, the role of autophagy in cancer is controversial, with both tumor-promoting and -inhibiting roles in leukemia suggested.^12,13^

It is well accepted that transformation events leading to AML may occur at the stem or progenitor cell stage. Hematopoietic stem cells (HSCs) strike a fine balance between quiescence, self-renewal and differentiation. When this balance is perturbed, the consequences may include biased differentiation and/or hematopoietic malignancies. In steady-state hematopoiesis, the majority of HSCs are quiescent.^14^ Quiescent cells are particularly hardy, able to survive long periods of metabolic stress. HSCs downregulate protein synthesis and activate pathways that sustain them during periods of non-division.^15^ Autophagy may be required for maintenance of the long-lived HSC, as their slow turnover prevents the dilution of damaged macromolecules to daughter cells, similar to a postmitotic neuron or cardiomyocyte.^16^ Moreover, autophagy controls mitochondrial quality.

In this study, we find autophagy levels highest in the most immature human and mouse hematopoietic stem and progenitor cells (HSPCs). Monoallelic loss of a key autophagy gene in a mouse AML model was sufficient to increase proliferation under metabolic stress *in vitro*, which was dependent on glycolytic metabolism, and led to leukemic progression *in vivo*. Combined with our findings of decreased autophagy in human AML patient blasts, these results suggest that dysregulated autophagy function may facilitate aberrant proliferation during AML development.

## Results

### Human and mouse HSPCs have high basal levels of autophagy

**Figure 1:**
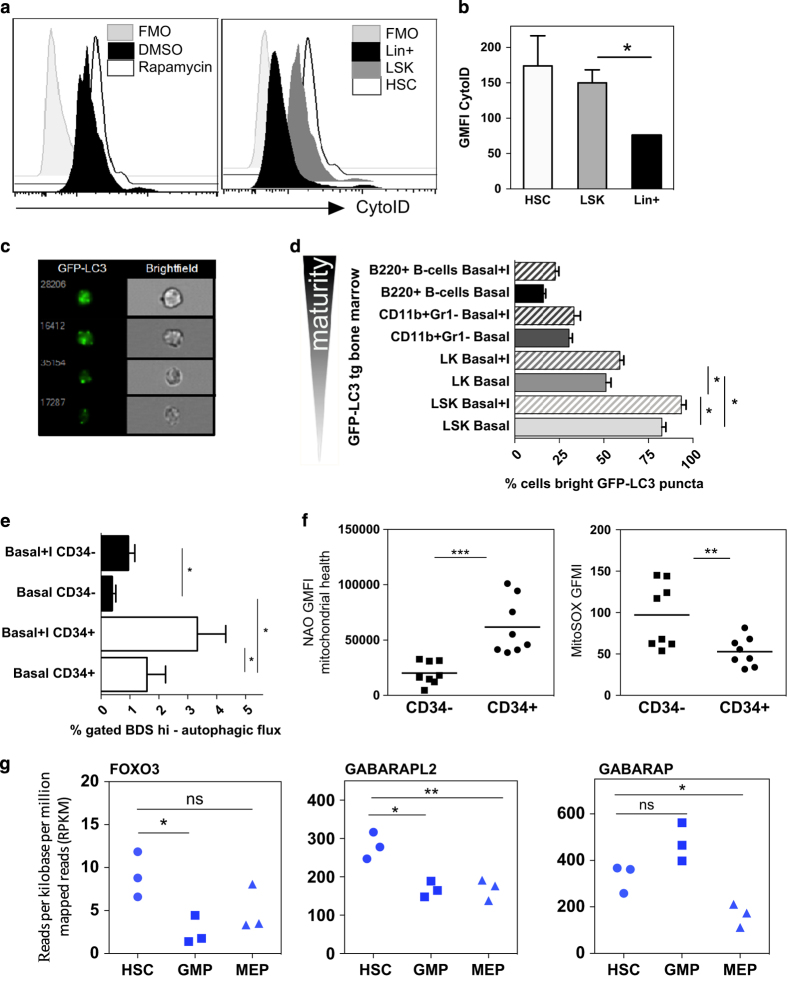
The autophagic flux is high in human and murine HSPCs. (**a**) Representative histograms of CytoID staining of indicated mouse bone marrow populations and indicated treatment, quantified as GFMI in (**b**) *n*=2, *t*-test. Rapamycin serves as control inducing autophagy. (**c**) Representative images and (**d**) quantification of *ex vivo* LC3-GFP puncta in LSK or LK HSPCs, CD11b^+^Gr1^−^ (neutrophil) or B220^+^ (B cell) BM populations from transgenic GFP-LC3 mice (‘+ I’ indicates 2 h E64D/PepA lysosomal inhibitor treatment), using Image Stream assessment of % cells with four or more bright puncta (*n*=4). (**e**) Frequency of CD34^+^ and CD34^−^ human BM mononuclear cells in LysoID/LC3 double-positive population with high LC3/LysoID BDS (Bright Detail Similarity; 20 000 cells analyzed per sample, *n*=6 healthy donors, Wilcoxon test *P<0.05 comparing populations within donors). (**f**) Geometric mean fluorescent intensity (GMFI) of nonyl-acridine orange (NaO, mitochondrial health) and (**c**) mitochondrial ROS (MitoSOX) staining in CD34^+^ and CD34^−^ human BM cells. (**g**) Gene expression (RNAseq analysis) of human HSC, GMP and MEP, *n*=3 healthy donors. All bar graph values are mean values± S.E.M.; *P*-values are obtained with Mann–Whitney test (*P<0.05, **P<0.01, ***P<0.001), unless otherwise stated.

We measured autophagic flux in mouse HSCs using three techniques (a) CytoID, an autophagosome- and autolysosome-pH-specific dye that can be directly detected by fluorescence intensity using conventional flow cytometry, (b) number of LC3-GFP puncta from transgenic mice by *imaging flow cytometry* and (c) number of endogenous LC3 puncta by *imaging flow cytometry*. For the latter two techniques imaging rather than intensity measurement is required to detect LC3 fluorescence, as upon autophagy induction LC3 relocalizes to the autophagosomal membranes from the cytoplasm.^17^ CytoID showed highest basal levels of autophagy in HSCs (Figures 1a and b). As Warr *et al.*^18^ demonstrated that mouse HSCs robustly induce autophagy after *ex vivo* cytokine withdrawal using LC3-based techniques, but failed to detect increased basal levels in murine HSCs, we next measured autophagic flux in bone marrow (BM) from GFP-LC3 mice *ex vivo* by *imaging flow cytometry* to quantify LC3-GFP puncta. This revealed significantly higher GFP-LC3 puncta levels in the Lin^−^Sca1^+^cKit^+^ (LSK) multipotent progenitor population, which are enriched for HSCs, relative to both the Lin^−^Sca1^−^cKit^+^ (LK) oligopotent progenitors and mature hematopoietic lineages (Figures 1c and d). Treatment with lysosomal inhibitors was performed to confirm the positive autophagic flux.^19^ We finally confirmed this result by quantifying endogenous LC3 puncta in wild-type (WT) C57BL/6 mice (Supplementary Figure S1A).

We next measured the autophagic flux in enriched human HSPCs using the imaging flow cytometry technique (Supplementary Figure S1B and C). We found that CD34^+^ HSPCs showed relatively higher basal autophagy levels than more mature CD34^−^ BM cells. Increased autophagic flux was confirmed by lysosomal inhibition (Figure 1e). The CD34^+^ HSPCs also showed improved mitochondrial health as measured by nonyl-acridine orange and reduced levels of mitochondrial reactive oxygen species (ROS) compared with the mature CD34^−^ cells (Figure 1f), maybe as a consequence of enhanced mitochondrial quality control by mitophagy. These findings were corroborated by RNAseq analysis of autophagy-related genes in human HSCs and granulocyte–macrophage progenitors (GMPs). A significant increase in expression was found for *FOXO3*, confirming data by Warr *et al.*^18^ in mice and for the two Atg8 homologs *GABARAPL2* and *GABARAP* (Figure 1g). These were the only autophagy-related genes found to be differentially expressed between HSCs and progenitors.

Overall, these results show that basal autophagy levels correlate with immaturity in human and murine hematopoietic cells compatible with an important role of autophagy in HSC function.

### Autophagy levels are decreased in primary human AML blasts, both transcriptionally and functionally

AML is characterized by usually heterozygous clonal chromosomal abnormalities.^20^ Our *in silico* analysis found multiple key autophagy genes^21^ within chromosomal regions commonly heterozygously deleted in AML^22–24^ (Table 1), in particular within regions often lost in complex karyotype AML. It is important to note that these chromosomal regions encompass many genes, and autophagy gene locations were often within regions more variably lost in patients.^25^ However, the presence of autophagy genes in the deleted regions was more frequent than expected by chance (*P*=0.039, Fisher’s exact test). These deletions were accompanied by decreased mRNA expression of multiple autophagy genes, in particular *Atg12*,^24^ a gene previously identified along with lysosomal gene *LAMP1* as downregulated in therapy-related MDS cases at the time of AML transformation.^26^

**Figure 2:**
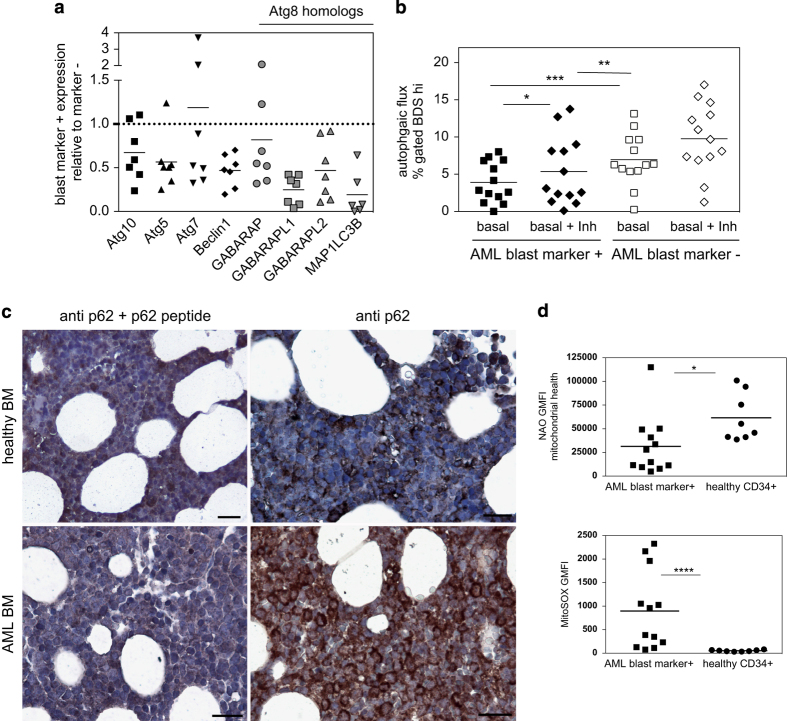
Decreased autophagy in human AML blasts. (**a**) Expression of autophagy genes in blast marker-positive BMNCs from AML patients relative to blast marker-negative cell expression (value for each donor represents the mean from three independent sorts, measured in triplicate by Fluidigm, normalized to GAPDH). (**b**) Frequency of blast marker-positive or -negative cells from AML BMNCs with high LC3/LysoID colocalization (BDS) under basal conditions or after E64D/Pepstatin A treatment (Basal+Inh; *n*=13, Wilcoxon test comparing populations within donors *P<0.05, **P<0.01, ***P<0.001). (**c**) Representative immunohistochemistry of p62 in bone marrow trephine samples from AML patients (*n*=8 donors) or healthy controls (*n*=4 donors) either with antibody pre-incubated with blocking peptide or with antibody alone (scale bar 50 *μ*m). (**d**) The geometric mean fluorescence intensity of nonyl-acridine orange (NaO) and ROS (by MitoSOX) of blast marker-positive BM cells from AML patients compared with CD34^+^ cells from healthy BM donors (Mann–Whitney test).

We next sought to examine the status of the autophagy pathway in human AML. Using AML BM samples, we sorted the populations identified by the dominant expanded blast markers in each patient (CD34, CD33, CD13 or combination, see Supplementary Figure S1D) and examined the expression of the key autophagy genes using the Fluidigm dynamic array. We found decreased expression of autophagy genes in the dominant AML blast cell population of the majority of donors tested when compared with the blast marker-negative population (Figure 2a). Human *Atg8* homologs, including *GAPARAPL1*, *GABARAPL2* (GATE-16) and *MAP1LC3B*, were particularly affected. There was no clear correlation between expression levels and available genetic/prognostic information.

To determine whether autophagy was functionally affected in human AML, we quantified autophagy in AML blasts using imaging flow cytometry as previously described^19^ and shown in Supplementary Figure S1B. This being the only technique applicable for such low number of cells, as both LC3 western blot and electron microscopy require cell sorting. We found that the expanded AML blast population had significantly lower autophagy flux than the remaining, blast marker-negative BM cells (Figure 2b). We also found that autophagy levels in blast marker+ AML cells decreased compared with total CD34+ HSPCs from healthy donors (Supplementary Figure S1D). As expected, chloroquine abolished the colocalization of LC3 and lysosomal marker in both populations, as it inhibits lysosomal fusion.

To further confirm the low autophagy phenotype, we examined levels of p62, a molecule that targets cargo for autophagic degradation, in AML BM trephine samples by immunohistochemistry. p62 is degraded with its cargo, thus accumulating when autophagy is decreased.^27^ We found widespread positive p62 staining in seven/eight AML BM samples, compared with zero/four healthy controls (Figure 2c), although we cannot exclude that increased transcription of p62 contributed to this.^28^ It is widely documented that autophagy controls ROS and mitochondrial health in hematopoietic cells.^29,30^ Accordingly, AML blasts showed decreased mitochondrial health and increased oxidative stress (Figure 2d).

### Identical phenotypes for *Atg5* and *Atg7* deficiency in HSCs *in vivo*

We have previously described the HSC, myeloproliferative and pre-leukemic phenotype of mice deficient for the essential autophagy gene *Atg7* (*VavAtg7*^*−/−*^).^30^ However, it was unclear whether this was because of Atg7’s role in autophagy.

We therefore knocked out *Atg5*, another key autophagy gene in mouse hematopoietic cells, using the Vav-Cre promoter (*VavAtg5*^*−/−*^). As observed in *VavAtg7*^*−/−*^ mice,^30^ all *VavAtg5*^*−/−*^ mice developed weight loss, lethargy along with lymphopenia (Supplementary Figure S2A and B) and progressive anemia (Supplementary Figure S2C and D) by 7–9 weeks of age. *Atg5*^*−/−*^ erythrocytes also had increased mitochondrial content and ROS (Supplementary Figure S2E and F). The LSK population was increased at 6 weeks in *VavAtg5*^*−/−*^ mice and were lost at 9 weeks (Supplementary Figure S2G), and mice died soon after (not shown). Also similar to the *Atg7* model, monocytes/macrophages (CD11b^+^F4/80^+,^ not shown) and neutrophils (CD11b^+^ Gr1^+;^ Supplementary FigureS3A and C) accumulated in *VavAtg5*^*−/−*^ mice in both spleen and peripheral blood. Histopathological assessment of BM cytospins confirmed the relative increase in neutrophils in blood and spleen (Supplementary Figure S3A and B) and in particular of immature myeloid cells in blood (Supplementary Figure S3D).^10^ Moreover, myeloid infiltrates were observed in hematopoietic and non-hematopoietic tissues (Supplementary Figure S3E), confirming severe myeloproliferation in the absence of *Atg5*.

### Heterozygous loss of *Atg5* in AML cells promotes proliferation *in vitro* and *in vivo*

**Figure 3:**
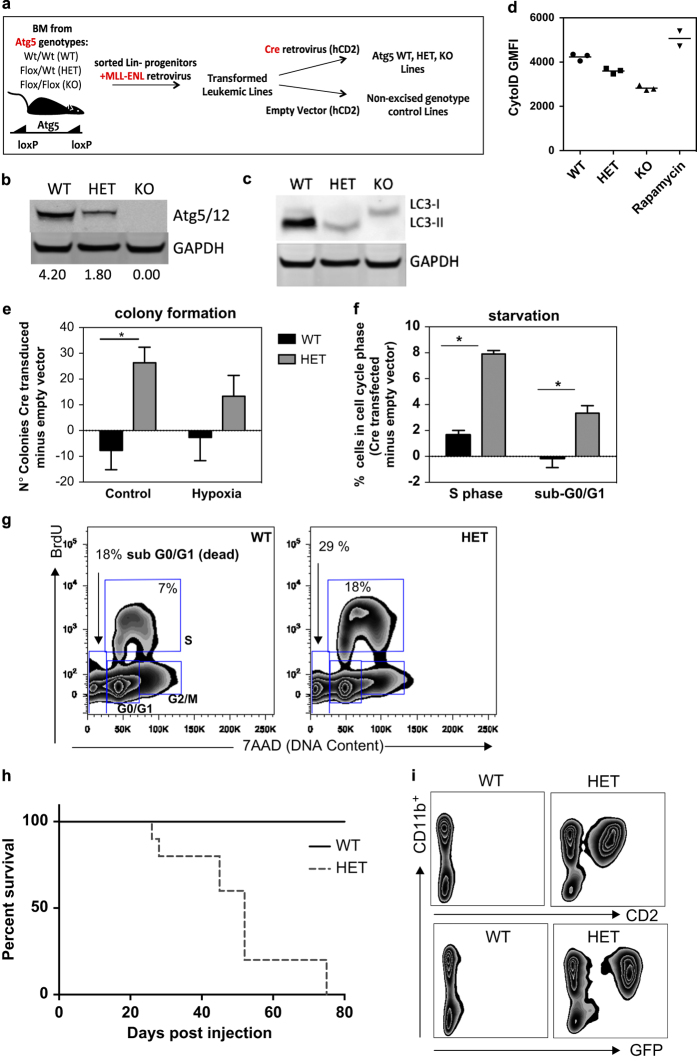
Heterozygous deletion of *Atg5* in a MLL–ENL AML model mediates increased proliferation and leukemogenic potential. (**a**) Generation of conditional *Atg5*^*−/−*^ (KO), *Atg5*^*+/−*^ (HET), *Atg5*^*+/+*^ (WT), empty vector or MLL–ENL transduced leukemic lines, in which Cre-expressing retrovirus was used to delete *Atg5* expression. (**b**) Western blot for Atg5 (Atg5 is typically only visible in Atg5/12 complex). (**c**) LC3 or GAPDH protein levels in protein lysates from leukemic lines. (**d**) Geometric fluorescence mean intensity of CytoID staining of MLL–ENL lines; Rapamycin treatment of WT lines serves as positive control. (**e**) difference in number of colonies formed in complete methylcellulose by 500 cells from leukemic lines following 72-h culture in normoxia or 0.1% oxygen (*n*=3 per line, representative of two experiments). (**f**) Quantification of BrdU plots from leukemic lines under starvation, gated as in **g**, showing difference in frequency of cells in the S phase or dead cells (sub-GO/G1; Cre-transduced subtract genotype empty vector frequency; *n*=5 cultures over three experiments) or (**g**) representative plots of BrdU incorporation against DNA content (7AAD intensity) delineating cell cycle stages and dead cells (sub-GO/G1) in leukemic lines starved for 18 h (reduced serum (2%) no growth factors). (**h**) Survival of sublethally irradiated C57BL/6 mice (2×3 Gy) intravenously injected with 1×10^6^ primary leukemic cells with *Atg5*^*wt/wt*^ or *Atg* ^*fl/wt*^ genotypes transduced with CRE-IRES-GFP retrovirus, sorted on GFP expression pre-injection; 10 mice per genotype were scored regularly and killed on development of clinical symptoms with pre-determined score threshold. All bar graph values are mean±S.E.M.; *P*-values are obtained with two-tailed *t*-test (*P<0.05), unless otherwise stated. See also Supplementary Figure S4 for normoxia (Supplementary Figure S4A) and hypoxia (Supplementary Figure S4B).

As our previous studies suggested that accumulated pre-leukemic Atg7-deficient cells did not harbor typical AML deletions or translocations and were not transplantable,^30^ we next asked whether autophagy has an impact on tumor growth in a mouse leukemia model with an existing translocation. We chose the translocation MLL–ENL as this fusion develops into an aggressive, fully penetrant AML *in vivo*, demonstrating that the engineered translocation can successfully model AML.^31^ Because deletion of autophagy genes is detrimental to untransformed hematopoietic precursors,^30^ we introduced the MLL–ENL fusion gene into non-excised *Atg5*^*wt/wt*^, *Atg5*^*fl/wt*^ and *Atg5*^fl/fl^ hematopoietic progenitor cells, followed by retroviral transduction with Cre to obtain *Atg5* WT, HET and KO lines, respectively (depicted in Figure 3a).^32,33^ Genotypes of the lines were confirmed using PCR and immature myeloid phenotype via CD11b, Gr1 and cKit expression (not shown). Expression of the Atg5 protein in complex with Atg12 was fully lost in KO lines and partially lost in the *Atg5* HET line (Figure 3b). We next assayed autophagy levels by LC3-II western blot (Figure 3c) and staining with CytoID for autophagosomes and autolysosomes (Figure 3d). Both techniques confirmed an intermediate autophagy defect in *Atg5* HET lines and a complete loss of autophagy in KO lines. However, *Atg5* KO ENL–MLL cell lines showed substantial cell death, preventing meaningful interpretation of subsequent data. Together with the fact that heterozygous loss of an autophagy gene is the most relevant genotype for AML, we focused on the *in vitro* and *in vivo* analysis on the HET genotype. However, the phenotypes of heterozygous *VavAtg5*^*+/−*^ and *VavAtg7*^*+/−*^ mice were identical to WT and showed no clinical symptoms even at 18 months of age (data not shown), suggesting that a primary oncogenic event such as MLL–ENL fusion is necessary to reveal this intermediate phenotype.

First we tested proliferation by examining MLL–ENL lines under normal conditions and under tumor stress conditions: nutrient/growth factor withdrawal and hypoxia. More colonies were counted in control conditions and hypoxia in the HET MLL–ENL line as compared with WT (Figure 3e). Analyzing short-term BrdU incorporation *versus* DNA content (7AAD), the HET lines displayed a proliferative advantage (S phase cells) following culture in withdrawal conditions (Figure 3f with example of gating in G, hypoxia and normoxia Supplementary Figure S4A and B). In summary, a partial deficiency in autophagy leads to increased proliferation in MLL–ENL leukemic lines *in vitro*. To assess the impact of *Atg5* loss on leukemia formation *in vivo*, primary *Atg5* WT and HET MLL–ENL leukemic cells were injected into sublethally irradiated hosts. At a time point at which 0/10 mice injected with the *Atg5* WT ENL–MLL lines had neither developed clinical symptoms nor signs of leukemia, 10/10 mice injected with HET *Atg5* MLL–ENL lines had to be killed because of typical leukemic clinical symptoms (Figure 3h). Representative FACS analysis showed the evidence of development of a biphenotypic CD2^+^CD11b^+^ and GFP^+^ leukemia (Figure 3).

### Altered metabolism and growth pathways upon AML development following heterozygous loss of *Atg5*

**Figure 4:**
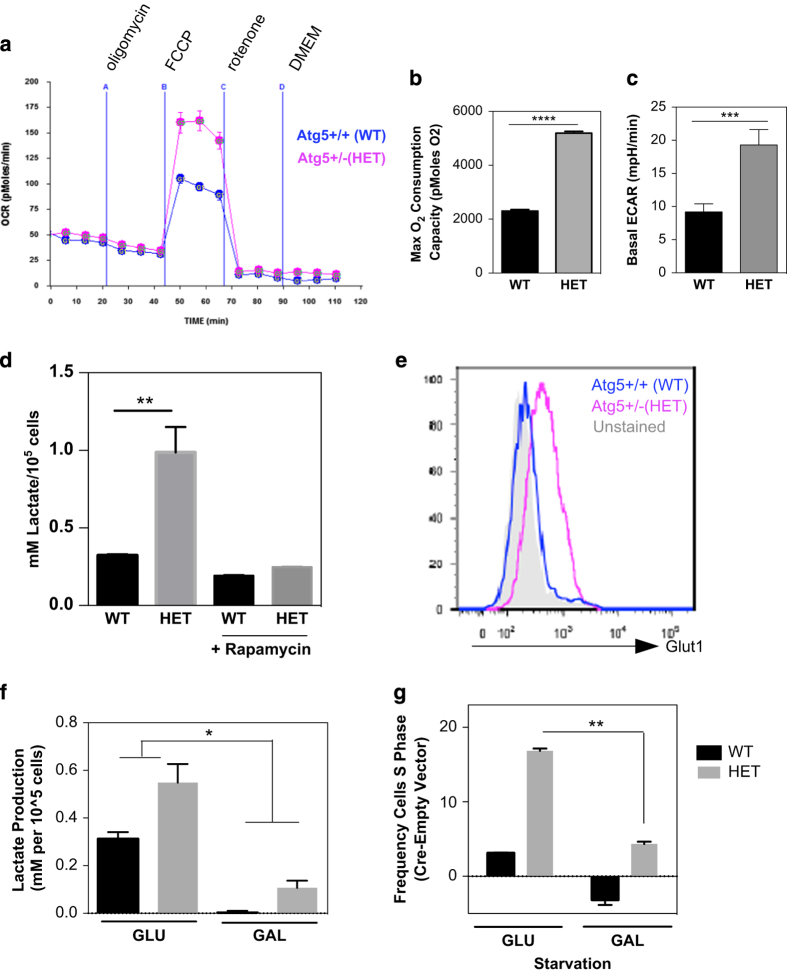
Heterozygous loss of *Atg5* causes increased glycolytic dependence. (**a**) OCR with (**b**) quantification after addition of FCCP and (**c**) basal ECAR of 280 000 cells from MLL–ENL cell lines assayed on the Seahorse Bioanalyzer with addition of mitochondrial inhibitors: (A) Oligomycin 400 nM; (B) FCCP, 400 nM; (C) Rotenone 1 µM; (D) media only (mean±S.D., five cultures assessed per genotype, representative experiment of two). (**d**) Enzymatic lactate assessment in supernatant of MLL–ENL lines after 48-h culture. (**e**) Histogram of Glut1 fluorescence intensity of MLL–ENL cell lines. (**f**) Enzymatic assessment of MLL–ENL line lactate production during culture with 20 mM glycolysis inhibitor galactose (GAL) or 20 mM glucose (GLU). (**g**) Frequency of cells in the S phase in Cre-transduced lines after culture with either 20 mM glucose or galactose, first for 24 h in complete media (10% FCS and cytokines) then under starvation conditions for 18 h (2% serum, no cytokines; *n*=2, mean±S.E.M.). *P*-values were obtained with two-tailed *t*-test (*P<0.05, **P<0.01, ***P<0.001, ****P<0.0001), unless otherwise stated.

Cancer cells convert glucose into lactate via aerobic glycolysis (termed the ‘Warburg effect’) to meet energetic demands and support proliferation.^34^ To test whether increased proliferation was accompanied by a shift toward glycolytic metabolism, we analyzed the oxygen consumption rate (OCR) and lactate production (extracellular acidification rate; ECAR) of the MLL–ENL cell lines using the Seahorse analyzer. Although both ENL–MLL lines displayed significant glycolysis, as previously reported,^35^ the *Atg5* HET cells displayed increased spare (unused) respiratory capacity (OCR after FCCP treatment; Figures 4a and b) and elevated basal ECAR (Figure 4c) compared with control lines. This glycolytic phenotype was confirmed by increased enzymatic quantification of lactate. Autophagy induction by Rapamycin inhibited glycolysis in both genotypes, as expected, and restored HET lactate production to levels comparable to the WT line (Figure 4d). Increased staining for the main glucose transporter Glut1 on the HET line confirmed increased glycolysis (Figure 4e). Next, we asked whether the HET line relied more on glycolysis for proliferation. Owing to ATP use for galactose processing, conversion of galactose to pyruvate via glycolysis generates no net ATP; therefore, cells must switch to oxidative phosphorylation to meet energy needs.^36^ As expected, galactose cultured MLL–ENL lines displayed minimal lactate generation (Figure 4f). Although cell death was unaltered in galactose (not shown), the HET cells showed a small but significant reduction in proliferation (Figure 4g). This suggests that increased proliferation following autophagy reduction in the HET is dependent on glycolytic metabolism, indicating that the increased glycolysis might at least partially cause the increased proliferation.

## Discussion

Autophagy genes, including *Atg5,* have been characterized as haploinsufficient tumor suppressors lost in multiple tumor types.^12,37^ Decreased levels of Atg5 have been associated with reduced progression-free survival and increased proliferation in melanoma.^38^ However, autophagy in AML biology has been relatively uninvestigated. Our findings are compatible with reduced expression levels of *Atg8* homologs in AML blasts, agreeing with a recent study across AML subtypes.^39^

We conclude that decreased autophagy could contribute, via increased proliferation, to leukemic development. These results agree with a recent study of *Atg7* deletion in pancreatic cancer to suggest deficient autophagy may augment tumor activity and metabolism.^40^ In that study and others, it appears that apoptotic impairment, for example, via p53 deletion, may be vital to turn pro-proliferative effects of *Atg* gene deletion into malignant benefits.^40,41^ As apoptosis is affected by MLL–ENL,^35,42^ our results support this hypothesis. However, previous findings were largely obtained in mouse cancer models. Here, we found that the majority of human AML donors measured retained low levels of autophagy activity and typically only display heterozygous Atg deletions.

Enhanced proliferation and aerobic glycolysis are common features of cancers, including AML.^43^ In addition to maintaining healthy mitochondria, macroautophagy may control glycolysis via the delivery of glycolytic enzymes to the lysosomes. Indeed, chaperone-mediated autophagy was shown to degrade the M2 isoform of pyruvate kinase.^44^ A third mechanism would be the degradation of free fatty acids (lipophagy) required to fuel the mitochondrial *β*-oxidation.^45^ It is possible to envisage that increased ROS may also have an impact on glycolysis.

In line with our data are the findings of a recent study showing that deficiency in PINK1, a kinase that controls mitophagy, sustains cell proliferation by re-programming glucose metabolism through HIF1.^46^

Here we found that physiological autophagy levels *ex vivo* were highest in immature hematopoietic HSPC populations. A similar pattern was observed during differentiation of cultured human stem cells.^47^ A recent study by Warr *et al.*^18^ highlighted the high autophagy capacity of HSC and its importance in HSC stress response; differences in the basal autophagy that Warr *et al.* measured in multipotent cells could reflect the cytokine-replete culture conditions used in many baseline controls for that study.

We predict that further investigation of the mechanistic interplay between autophagy, energy metabolism and cell cycle status in leukemic and stem cells will lead to a better mechanistic understanding of stem cell fate decisions and the discovery of new therapeutic strategies in anticancer therapy.

## Materials and methods

### Flow cytometry

Flow cytometry experiments were performed on LSRII (Becton Dickinson, Oxford, UK) or CyAn (Beckman Coulter, High Wycombe, UK) instruments, unless otherwise stated. Data were analyzed with FlowJo 8.8.6 for Mac (Tree Star Inc., Ashland, OR, USA). Single-cell suspensions of human bone marrow mononuclear cells (BMNCs) were rested overnight for 24 h in IMDM 20% FCS before staining; single-cell suspensions of murine BM, spleen and peripheral blood were stained immediately after harvesting. Fc blocking and live/dead reagents were used for all appropriate experiments. Absolute cell numbers in the peripheral blood were quantified using TruCount tubes (Becton Dickinson). See Supplementary Tables 2–4 for antibodies and reagents used.

### CytoID assay

Autophagosomal content was quantified with the CytoID kit (Enzo Life Sciences, Exeter, UK). Murine BM was cultured in CytoID dye (1 : 3000) containing medium for 30 min according to the manufacturer's protocol. Cells were stained with surface antibodies to identify HSC (Lin^−^Sca1^+^cKit^+^CD150^+^CD48^−^) and LSK (Lin^−^Sca1^+^cKit^+^) populations and were analyzed using flow cytometry. Rapamycin and Wortmannin were used as internal controls.

### Imaging flow cytometry (image stream) and autophagy assay

After material preparation as above, where applicable samples were suspended <1×10^6^ cells/ml in either 1x HBSS 4.2 mM sodium bicarbonate (starvation media, only where stated) or normal growth media (IMDM 10% FCS for primary cells and as stated for cell lines) with 10 *µ*g/ml E64D/Pepstatin A (Enzo Life Sciences), 1 *µ*M Rapamycin (Sigma, Gillingham Dorset, UK), 20 *µ*M Chloroquine (Sigma) or 100 nM Wortmannin (Sigma) for 2 h. Image stream measurement of autophagy was performed as previously described,^48^ samples were acquired on the Image Stream 100 or Image Stream X (Amnis, Watford, UK), data obtained were analyzed with the IDEAS 4.0.735 software (Amnis) for LC3/LysoID Bright Detail Similarity (that is, the similarity between their fluorescent signals). Cells from LC3-GFP transgenic mouse, previously published,^49^ were acquired on the IS100 and analyzed for spot count on a LC3 peak mask with a spot to cell background ratio of 2.5 or 3.5. See Supplementary Tables 2–4 for antibodies and reagents used.

### Human BM

Healthy human BMNCs were obtained from Lonza (2M-125C, Verviers, Belgium). AML and MDS BMNCs were obtained from MDSBio (experiments approved by local REC, REC# 11/H0605/4 with informed consent).

### RNA-seq

Human BM was sorted for HSC (Lineage^−^CD34^+^CD38^−^CD90^+^CD45RA), GMP (Lineage^−^CD34^+^CD38^+^CD123^+^CD45RA^+^) and MEP (Lineage^−^CD34^+^CD38^+^CD123^−^CD45RA^−^) populations; RNA was extracted and RNAseq analysis was performed; reads per kilobase per million mapped reads for autophagy genes was calculated using standard techniques.^50^

### Fluidigm gene expression analysis

AML BMNCs were sorted on selected blast surface marker expression (Supplementary Figure S6); 3×200 cells/population per donor. RNA was extracted, selected autophagy and metabolic genes amplified and analyzed for expression using a dynamic 48×48 array (Biomark Fluidigm) as previously described.^51^ See Supplementary Table 1 below for Taqman gene expression assays.

### P62 immunohistochemistry

Serial 4 *µ*m sections of BM trephine samples taken from AML patients or lymphoma patients with no BM involvement were stained with anti-human p62 (Enzo Life Science BML-PW9860) using the Dako (Cambridge, UK) EnVison+ Dual Link System (K4065) and standard techniques. Synthetic peptide (HCPPEADPRLIESLSQMLSMGFSDEGGWLTRLLQTKNYD-IGAALDTIQYSKH-OH) pre-incubated with primary antibody at 400x molar excess was used as negative control.

### Mice

*Vav Atg5/7*^*−/−*^ mice were on the C57BL/6 background, and bred and housed in the Department of Biomedical Services, University of Oxford, in individually ventilated cages. Age- and sex-matched mice were used at the age of 6–9 weeks for all experiments, unless stated otherwise. VaviCre−; Atg5/7^fl/fl^ and VaviCre^+^; Atg5/7^fl/wt^ littermates were used equally as controls. Vav-Atg7 breeding and genotyping are described previously.^30^ Atg5^fl/fl^ mice were crossed to VaviCre mice to obtain VavAtg5. Atg5 wt and flox genotyping were performed separately.^52^ All animal experiments were approved by the local ethical review committee and were performed under a UK home office license (PPL 39/2809).

### Histology

BM was aseptically collected from 7-week-old VavAtg7^*−/−*^ mice and littermate controls, and stained using APC anti-mouse Gr1 and PE anti-mouse CD11b and were then sorted using flow cytometry. Histology was performed as previously described.^30^

### BM cytospin

Filter cards were moistened with PBS before spinning. Overall, 5000 BM cells from 6-week-old VavAtg5−/− mice or WT littermates were resuspended in 200 *µ*l of PBS/20% FCS and pipetted onto the filter cards and then centrifuged with low acceleration, 1000 r.p.m. for 10 min before drying overnight. Slides were soaked in May–Grünwald for 5 min, washed with DI water, before being transferred to Giemsa for 20 min, and washed with DI water again. Slides were then left to dry before mounting with Pertex. Slides were inspected with an uninverted microscope and 100 cells per slide were morphologically scored as: Neutrophil, Immature Myeloid, Lymphocyte, Erythrocyte.

### MLL–ENL line generation

BM from *Atg5* fl/fl, *Atg5* fl/wt and *Atg5* wt/wt (Bl6) mice (three per genotype) was extracted, crushed and pooled by genotype. Lineage cells were depleted and Lin-negative cells transduced with constitutive MLL–ENL, as previously described.^32,33^ The resulting lines were transduced with pMSCV-IRES-CD2, containing the tailless human CD2 cDNA^53^ either alone (empty vector) or with additional iCre^54^ and grown in RPMI, 10% FCS, 2 mM L-Glutamine, penicillin/streptomycin, 50 *μ*M 2-mercaptoethanol, 100 ng/ml mSCF, 10 ng/ml mIL-3 and 10 ng/ml mIL-6.

### Immunoblot

Protein was extracted using RIPA buffer with supplemental protease Inhibitors (Roche, Welwyn Garden City, UK). Protein content was normalized and samples boiled with 4x LDS sample buffer (Invitrogen, Lutterworth, UK) and 20x Reducing agent (Invitrogen) and then run on a 4–12% Bis-Tris gel (Invitrogen). Proteins were transferred to PVDF membranes (GE Healthcare, Little Chalfont, UK) and blocked overnight in blocking buffer (TBS-T 5% skimmed milk). Primary antibodies (all Cell Signaling (Hitchin, UK) except anti-Atg5 from Novus and anti-LC3 from MBL-Int. (Woburn, MA, USA)) were incubated at 1 : 1000 in blocking buffer, at 4 °C overnight. Secondary antibodies were Goat *α*-rabbit 800 CW or Goat *α*-rat 680LT (Licor, Cambridge, UK). Blot detection was carried out using Odyssey CLx Infrared Imaging System.

### BrdU analysis

For multiday BrdU incorporation analysis, cell lines were cultured for 48 h with 10 *µ*M BrdU and then stained for incorporation as per the BrdU Flow Kit (Becton Dickinson); cell cycle kinetics were analyzed following 40 min BrdU pulse. MLL–ENL line starvation was performed overnight with 2% FCS and no cytokines; hypoxia treatment was 48 h in 0.1% oxygen.

### Seahorse and metabolic assessments

In all, 2.8×10^5^ cells per well were adhered to microplates using Cell Tak (Becton Dickinson). Cells were washed and rested for 1 h in low-buffered bicarbonate-free DMEM (pH 7.4) before rates of glycolysis and oxidative phosphorylation were determined in 10 replicates for each genotype by measuring lactic acid release (ECAR) and OCR using an XF24 XF analyser (Seahorse Bioscience, Billerica, MA, USA) as previously described.^55^ Enzymatic lactate assessment used the Instruchemie kit (2864) in 96-well plate.

### Statistical analysis

Given prior experience with the VavAtg7 model, where we found 100% penetrance for the vast majority of phenotypes, we usually chose the number of WT and KO mice to be the minimum (*n*=4) that still gives statistical significance (*P*<0.05) using a Mann–Whitney test. The number of mice in all other animal experiments (BM chimera, MLL-ENL *in vivo* experiments) are shown or indicated. No animals or data were excluded. Animals were never randomized. Blind analysis was performed by the pathologist for the murine immunohistochemistry (Supplementary Figure S3E and cell counts on blood smears (Supplementary Figure S3D). The exact sample size (*n*), a description of the sample collection and the number of times an experiment has been replicated is described in the appropriate figure legends. Statistics analysis was performed using Graph Pad Prism 4, error bars represent S.E.M. and *P*-values calculated with a two-tailed Mann–Whitney test unless otherwise stated.

## Figures and Tables

**Table 1:** Autophagy genes are hemizygously deleted in human AML

*Chromosome lost (normally heterozygous)*	*Deleted regions*	*Autophagy genes*	*Autophagy gene locations (human genome, taken from Ensembl Genome Browser)*	*Frequency chromosome lost in complex karyotype AML; % (*n*=60, Rucker et al.*^26^)
5q	Chr 5: 79 741 823–159 483 514	*Atg10*	Chr 5: 81 267 844–81 572 241	77
		*Atg12*	Chr 5: 115 163 897–115 177 555	
7q	Chr 7: 107 570 049–158 821 424	*KLHDC10*	Chr 7: 129 710 350–129 773 596	45
		*PRKAG2*	Chr 7: 151 253 203–151 574 316	
		*Atg9B*	Chr 7: 150 709 302–150 721 586	
12p	Chr 12: 100 000–32 700 000*	*GABARAPL1*	Chr 12: 10 365 489–10 375 722	18
16q	Chr 16: 59 672 953–88 827 254	*GABARAPL2*	Chr 16: 75 600 249–75 611 779	32
		*MAP1LC3B*	Chr 16: 87 425 406–87 438 385	
17p	Chr 17: 1–8 353 903	*GABARAP*	Chr 17: 7 143 333–7 145 772	55

Correlation between the locations of autophagy pathway genes in the human genome, as compiled by Behrends *et al.*^21^ and chromosomal regions commonly deleted in AML patients.^22,24,56^ *P*=0.039 indicates the statistical probability of this number of core autophagy genes that are being deleted by chance, assuming independent gene locations, Fisher’s exact test.

## Abbreviations

AML: acute myeloid leukemia
BM: bone marrow
GMP: granulocyte–macrophage progenitors
ECAR: extracellular acidification rate
HET: heterozygous
HSC: hematopoietic stem cells
HSPC: hematopoietic stem and progenitor cells
KO: knock-out
LC3: microtubule-associated protein light chain 3
LK: Lin^−^Sca1^−^cKit^+^
LSK: Lin^−^Sca1^+^C-kit^+^
MDS: myelodysplastic syndrome
MLL–ENL: mixed lineage leukemia–eleven-nineteen leukemia
OCR: oxygen consumption rate
ROS: reactive oxygen species
WT: wild type.
