# Supplementary material for: Autophagy limits proliferation and glycolytic metabolism in acute myeloid leukemia
Source: Cell Death Discov. 2015 Aug 17;1:15008–. doi: 10.1038/cddiscovery.2015.8 (PMC4641322; doi:10.1038/cddiscovery.2015.8)
Supplement: Supplementary Figure 1 [file cddiscovery20158-s1.pdf]

Supplementary Figure 1

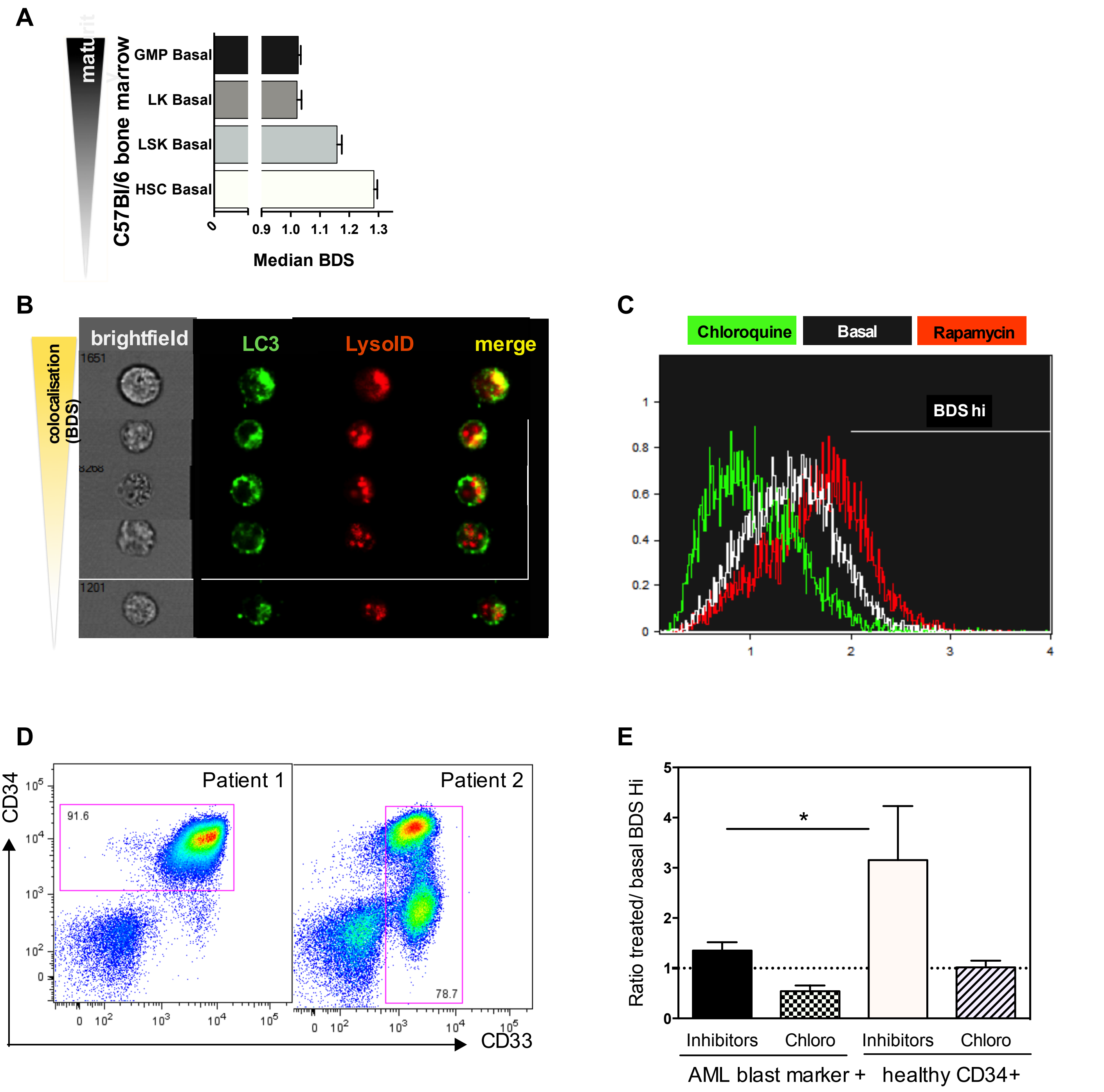

Figure S1 (**A**) Median LC3/LysoID BDS of murine HSC (Lin-Sca1+cKit+CD150+CD48-), LSK (Lin-Sca1+cKit+), LK myeloid progenitors (Lin-Sca1-cKit+) and GMP (Granulocyte-macrophage progenitor, Lin-Sca1-cKit+FcgRII/IIIhi) bone marrow populations (note that median BDS is employed rather than %BDShi due to HSC population rarity) (150,000 cells analysed per sample, n=3 mice, t-test $p < 0.05$) **(B)** Representative images of cells from Image Stream Flow Cytometry (10,000 cells analyzed per sample) sorted for increasing colocalization of LC3 and lysosomes (LysoID) (**C**) Representative histograms of autophagy quantification by LC3 and LysoID colocalization (Bright Detail Similarity, BDS) of 10,000 cells cultured in normal medium (basal) and with treatment indicated (Chloroquine, autophagy inhibitor; Rapamycin, autophagy inducer)**(D)** Acute myeloid leukemia patient immunophenotyping and blast marker gating, example dot plots for CD33/CD34 expression of bone marrow mononuclear cells from acute myeloid leukemia patients, showing donors where CD34 (left) or CD33 (right) best defines the blast population. The single or two-marker combination that represents the highest proportion of the blast population was chosen after evaluation of 5 blast markers and used for Image Stream and Fluidigm analysis (**E**) Ratio of cells treated with E64D/Peptsatin A or Chloroquine over untreated cells, gated on BDShi and CD33+ or CD34+ or CD13+ or a combination from AML donors, or CD34+ from healthy donors, t-test, $p<0.05$.
