# Supplementary material for: Autophagy limits proliferation and glycolytic metabolism in acute myeloid leukemia
Source: Cell Death Discov. 2015 Aug 17;1:15008–. doi: 10.1038/cddiscovery.2015.8 (PMC4641322; doi:10.1038/cddiscovery.2015.8)
Supplement: Supplementary Figure 2 [file cddiscovery20158-s2.pdf]

Supplementary Figure 2

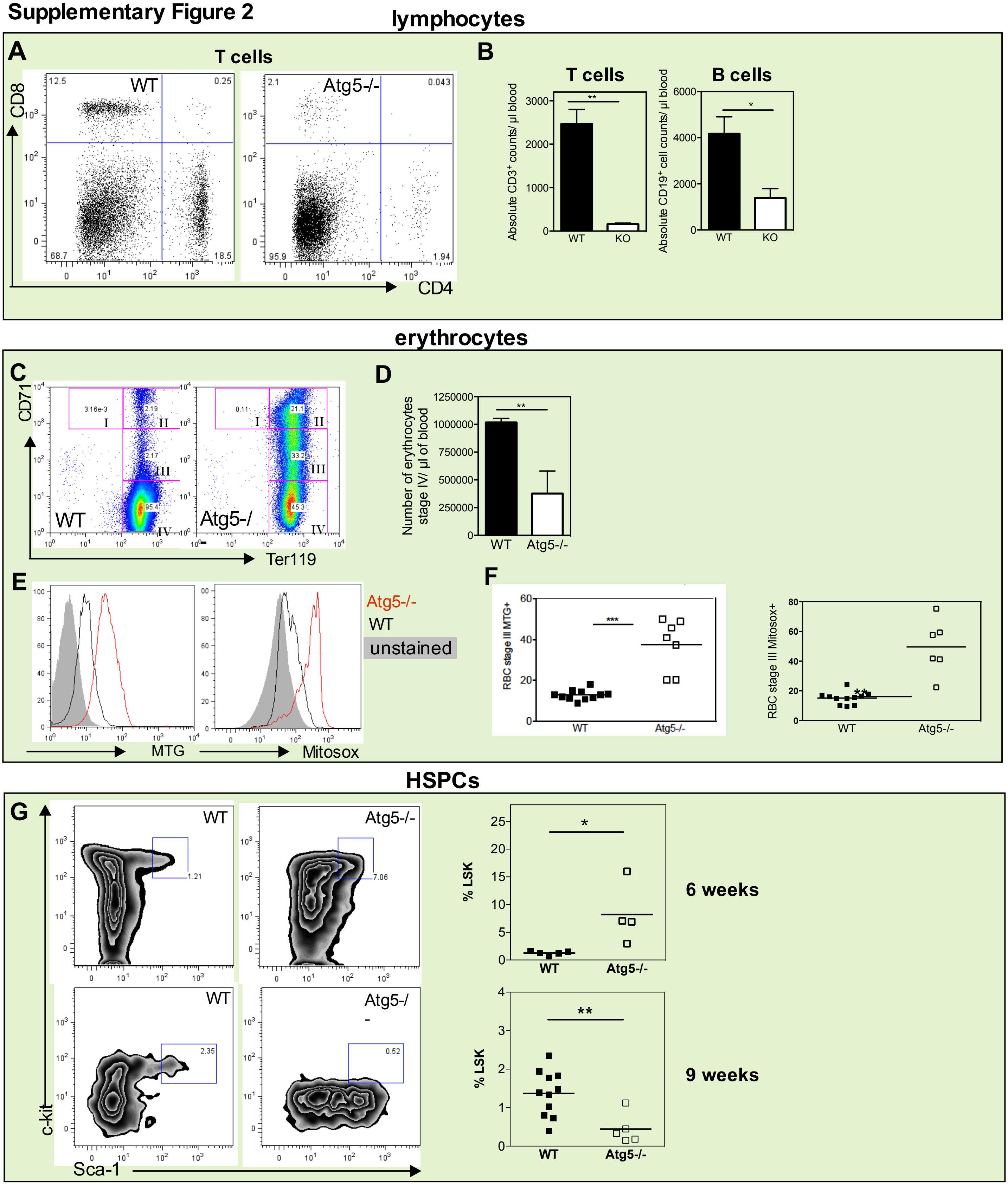


**Figure S2 VavAtg5$^{-/-}$ lymphoid and erythroid cells are decreased in number** (**A**) Representative CD4/CD8 FACS plots of VavAtg5$^{-/-}$ and WT littermate spleen at 6 weeks of age (**B**) Absolute blood counts of CD3$^{+}$ T cells and CD19$^{+}$ B cells in VavAtg5$^{-/-}$ and WT mice. (**C**) Representative FACS plots of VavAtg5$^{-/-}$ and WT blood at 6 weeks of age showing CD71/ Ter119 defined erythroid development stages I-IV. (**D**) Absolute count in 9 week old VavAtg5$^{-/-}$ and WT mice of stage IV erythroid cells in blood. (**E**) MitoTracker Green (MTG) intensity in stage III erythroid cells, with representative overlaid histograms for each genotype (top) and frequency of MTG positive cells (bottom). (**F**) Mitochondrial ROS (MitoSOX intensity) in stage III VavAtg5$^{-/-}$ and WT erythroid cells, with representative overlaid histograms for each genotype (top right) and frequency of MitoSOX positive cells (bottom right). (**G**) Frequency of LSK cells in BM from *VavAtg5*$^{-/-}$ or WT mice at 6 weeks of age (top) or 9 weeks of age (bottom). All bar graph values are mean ± s.e.m., statistics are from Mann Whitney tests; $p < 0.05$
