# Supplementary material for: Autophagy limits proliferation and glycolytic metabolism in acute myeloid leukemia
Source: Cell Death Discov. 2015 Aug 17;1:15008–. doi: 10.1038/cddiscovery.2015.8 (PMC4641322; doi:10.1038/cddiscovery.2015.8)
Supplement: Supplementary Figure 3 [file cddiscovery20158-s3.pdf]

Supplementary Figure 3

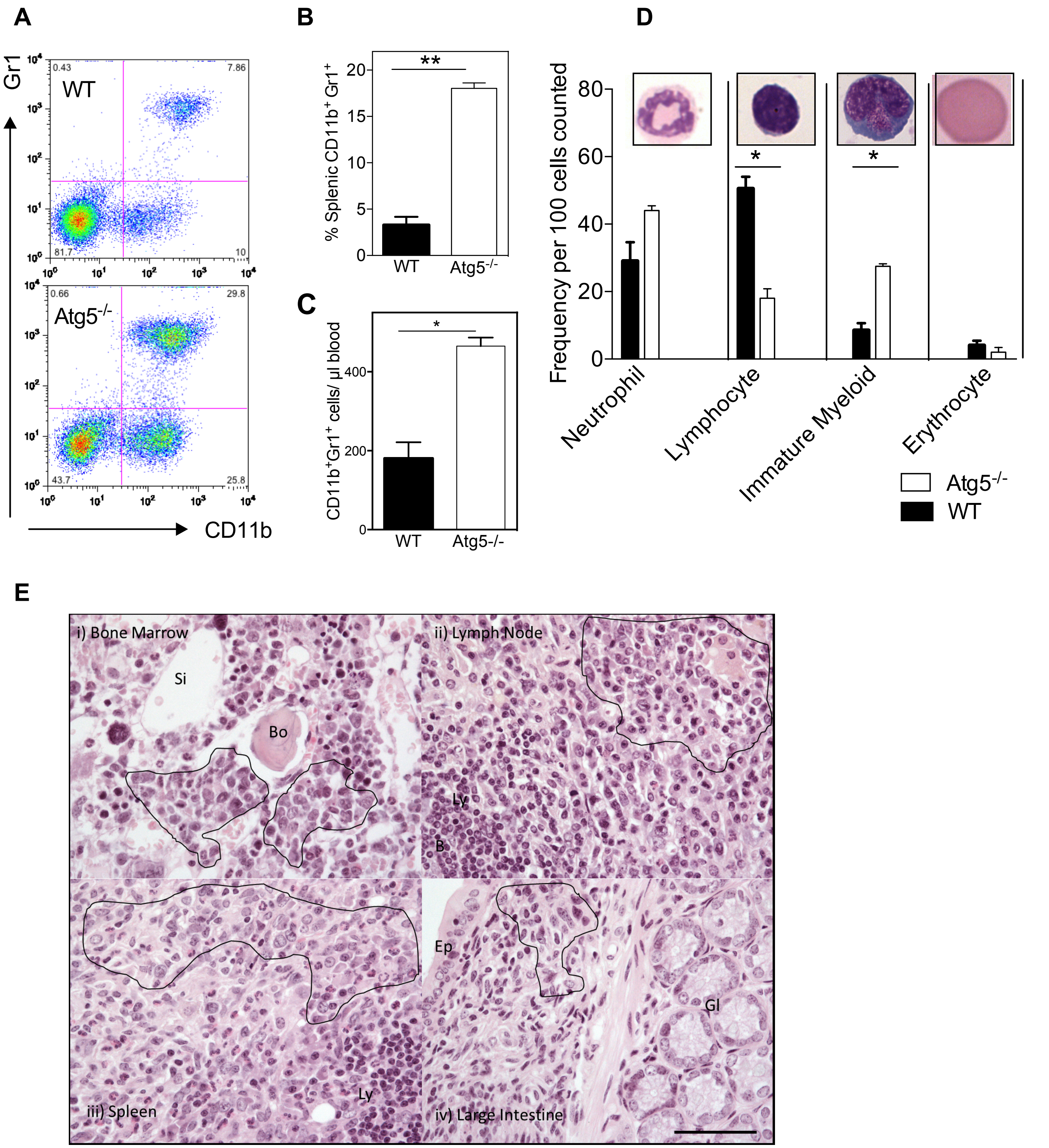

Figure S3 Autophagy deficient myeloid cells accumulate *in vivo* (**A**) Representative flow cytometry plots of CD11b and Gr1 expression in peripheral blood of 6 week old *VavAtg5*$^{-/-}$ mice or wild type (WT) littermate controls. (**B**) Frequency of CD11b^{+}Gr1^{+} cells among total splenocytes from *VavAtg5*$^{-/-}$ mice and WT mice (n=3, mean s.e.m.) and (**C**) absolute counts in blood. (**D**) Frequency per 100 cells of indicated cell types in cytospins of 6 week old *VavAtg5*$^{-/-}$ mouse bone marrow or that of WT littermate controls along with representative images of each cell type (counted in duplicate, mean s.d., representative of two time points). (**E**) Paraffin embedded tissue sections of *VavAtg5*$^{-/-}$ mice showing dysplastic infiltrates in BM (Bo-bone, Si-sinus), lymph nodes (Ly-lymphocyte), spleen and large intestine (Gl-gland, Ep-epithelium) (scale bar 50 µm all panels).
