# Supplementary material for: Autophagy limits proliferation and glycolytic metabolism in acute myeloid leukemia
Source: Cell Death Discov. 2015 Aug 17;1:15008–. doi: 10.1038/cddiscovery.2015.8 (PMC4641322; doi:10.1038/cddiscovery.2015.8)
Supplement: Supplementary Figure 4 [file cddiscovery20158-s4.pdf]

Supplementary Figure 4

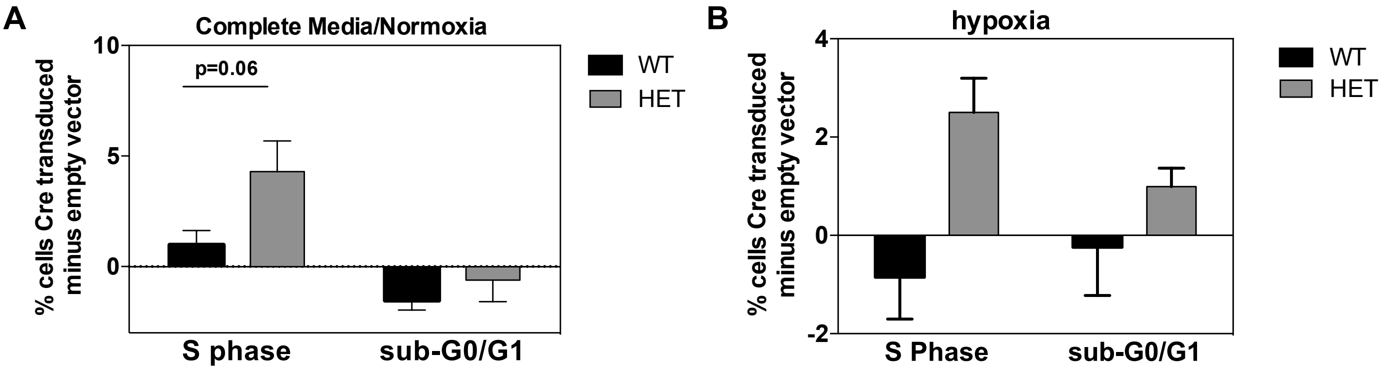

Figure S4 HET MLL-ENL display increased cell proliferation and apoptosis. Differences in frequency of cells in S phase or sub-G0/G1 (dead) and BrdU pulse (Empty vector substracted from Cre transduced for each relevant genotype) (**A**) after culture in complete media (10% FCS and cytokines) and normoxia for 40 hrs or (**B**) after 40 hrs in 0.1% oxygen; error bars represent SEM (n=5), unpaired t-test.
