# Supplementary material for: Autophagy limits proliferation and glycolytic metabolism in acute myeloid leukemia
Source: Cell Death Discov. 2015 Aug 17;1:15008–. doi: 10.1038/cddiscovery.2015.8 (PMC4641322; doi:10.1038/cddiscovery.2015.8)
Supplement: Supplementary Tables [file cddiscovery20158-s5.doc]

**SUPPLEMENTARY METHODS TABLES**

**Supplementary Table 1**: qRT-PCR Taqman probes (from Applied Biosystems)

| **Species** | **Target Gene** | **Probe ID** |
| --- | --- | --- |
| **Human** | GAPDH | Hs02758991_g1 |
| **Human** | Atg5 | Hs00169468_m1 |
| **Human** | Atg7 | Hs00883770_m1 |
| **Human** | Atg10 | Hs00919721_m1 |
| **Human** | Atg12 | Hs00740818_m1 |
| **Human** | Becn1 | Hs00186838_m1 |
| **Human** | GABARAP | Hs009258991_g1 |
| **Human** | GABARAPL1 | Hs00740588_mH |
| **Human** | GABARAPL2 | Hs00371854_m1 |
| **Human** | MAP1LC3B | Hs00917682_m1 |
| **Human** | Atg9A | Hs00225393_m1 |
| **Mouse** | GAPDH | Mm99999915_g1 |
| **Mouse** | Atg9A | Mm01264429_m1 |
| **Mouse** | Atg7 | Mm00512209_m1 |
| **Mouse** | Becn1 | Mm00517174_m1 |
| **Mouse** | Atg5 | Mm00504340_m1 |
| **Mouse** | Atg12 | Mm00503201_m1 |
| **Mouse** | Atg10 | Mm00470550_m1 |
| **Mouse** | HPRT | Mm01545399_m1 |
| **Mouse** | B2M | Mm00437762_m1 |
| **Mouse** | PDK1 | Mm00554300_m1 |
| **Mouse** | PDK2 | Mm00446681_m1 |
| **Mouse** | Hif1a | Mm00468869_m1 |
| **Mouse** | Pgm1 | Mm00804141_m1 |
| **Mouse** | PHGDH | Mm01623589_g1 |
| **Mouse** | Pgc1a | Mm01208835_m1 |
| **Mouse** | Fbp1 | Mm00490181_m1 |
| **Mouse** | HK3 | Mm01341942_m1 |
| **Mouse** | IDH1 | Mm00516030_m1 |
| **Mouse** | IDH2 | Mm00612429_m1 |
| **Mouse** | CDKN1C | Mm01272135_g1 |

**Supplementary Table 2**: Reagents used in mouse flow cytometry or imaging flow cytometry (antibodies are all anti-mouse)

| Name (clone) | Fluorochrome | Company |
| --- | --- | --- |
| **CD4 (RM-4.5)** | unconjugated or PE-Cy5 | eBioscience |
| **CD5 (53-7.3)** | unconjugated or PE-Cy5 | eBioscience |
| **CD8a (53-6.7)** | unconjugated or PE-Cy5 | eBioscience |
| **CD11b (M1/70)** | unconjugated, PE-Cy5, PE, eFluor 450 | eBioscience |
| **B220 (RA3-6B2)** | Unconjugated, PE-Cy5, eFluor 450 | eBioscience |
| **Ter119 (TER-119)** | Unconjugated, PE-Cy5, PE | eBioscience |
| **Gr1 (RB6-8C5)** | Unconjugated, PE-Cy5, FITC | eBioscience |
| **F4/80** | APC | eBioscience |
| **Goat anti-rat IgG (Poly 4054)** | Dylight 405 (secondary) | Biolegend |
| **Goat anti-rat IgG** | Cy5-R-PE (secondary) | Invitrogen |
| **CD34 (MEC14.7)** | PE | eBioscience |
| **CD71** | biotinylated | eBioscience |
| **CD117/c-Kit (2B8)** | APC-eFluor 780  APC | eBioscience  eBioscience |
| **Sca-1 (E13-161.7)** | Pacific Blue | Biolegend |
| **Sca-1 (D7)** | biotinylated | eBioscience |
| **Streptavidin** | PE  APC  PE-TexasRed | BD  eBioscience  BD |
| **CD150 (TC15-12F12.2)** | PE-Cy7 | eBioscience |
| **CD105 (MJ7/18)** | biotinylated | eBioscience |
| **CD48 (HM48-1)** | PE  APC | eBioscience  Biolegend |
| **CD16/32 (93)** | PE | eBioscience |
| **Fc blocking reagent (anti-mouse CD16/32) (93)** | N/A | eBioscience |
| **Annexin V** | APC | Biolegend |
| **Glut1** | PerCP | R&D Systems |

**Supplementary Table 3**: Reagents used in human flow cytometry or imaging flow cytometry (antibodies are all anti-human)

| Name (clone) | Fluorochrome | Company |
| --- | --- | --- |
| **CD34 (581)** | PE  FITC | Biolegend  BD |
| **CD33 (P67.6),** | eFluor 450 | eBioscience |
| **CD13 (WM15)** | PE | BD |
| **CD14 (TuK4)** | Pacific Blue | Invitrogen |
| **CD117 (104D2)** | PE | Biolegend |
| **Human Fc Blocking Reagent** | N/A | MACS Miltenyi Biotec |
| **BrdU Flow Kit** | APC | BD |

**Supplementary Table 4**: General reagents used in both human and mouse flow cytometry or imaging flow cytometry

| Reagent | Working concentration | Company |
| --- | --- | --- |
| **7AAD** | N/A | eBioscience |
| **Aqua Fluorescent reactive dye** | N/A | Invitrogen |
| **Violet Fluorescent reactive dye** | N/A | Invitrogen |
| **nonyl Acridine Orange** | 100nM | Invitrogen |
| **MitoSOX Red** | 5uM | Invitrogen |
| **MitoTracker Green** | 100nM | Invitrogen |
